# Hyperspectral machine-learning model for screening tea germplasm resources with drought tolerance

**DOI:** 10.3389/fpls.2022.1048442

**Published:** 2022-12-01

**Authors:** Sizhou Chen, Jiazhi Shen, Kai Fan, Wenjun Qian, Honglian Gu, Yuchen Li, Jie Zhang, Xiao Han, Yu Wang, Zhaotang Ding

**Affiliations:** ^1^ Tea Research Institute, Shandong Academy of Agricultural Sciences, Jinan, Shandong, China; ^2^ Tea Research Institute, Qingdao Agricultural University, Qingdao, Shandong, China; ^3^ Key Laboratory of Tea Biology and Tea Processing of Ministry of Agriculture, Anhui Agricultural University, Hefei, Anhui, China; ^4^ International Tea Science and Technology Innovation Institute, Nanjing Agricultural University, Nanjing, Jiangsu, China

**Keywords:** tea germplasm resources, hyperspectral imaging, machine learning, nondestructive testing, drought tolerance

## Abstract

Drought tolerance and quality stability are important indicators to evaluate the stress tolerance of tea germplasm resources. The traditional screening method of drought resistant germplasm is mainly to evaluate by detecting physiological and biochemical indicators of tea plants under drought stresses. However, the methods are not only time consuming but also destructive. In this study, hyperspectral images of tea drought phenotypes were obtained and modeled with related physiological indicators. The results showed that: (1) the information contents of malondialdehyde, soluble sugar and total polyphenol were 0.21, 0.209 and 0.227 respectively, and the drought tolerance coefficient (DTC) index of each tea variety was between 0.069 and 0.81; (2) the comprehensive drought tolerance of different varieties were (from strong to weak): *QN36, SCZ, ZC108, JX, JGY, XY10, QN1, MS9, QN38*, and *QN21*; (3) by using SVM, RF and PLSR to model DTC (drought tolerance coefficient) data, the best prediction model was selected as MSC-2D-UVE-SVM (R^2^ = 0.77, RMSE = 0.073, MAPE = 0.16) for drought tolerance of tea germplasm resources, named Tea-DTC model. Therefore, the Tea-DTC model based on hyperspectral machine-learning technology can be used as a new screening method for evaluating tea germplasm resources with drought tolerance.

## 1 Introduction

With global warming, drought has become one of the major natural disasters (Sharma and Kumar, 2005). This problem is becoming more and more serious (Farooq et al., 2012). It is reported that drought reduced tea production by 14-33%, resulting in 6-19% plant death (Cheruiyot et al., 2010). Under drought stress, the yield and quality of tea plants will decline, seriously restricting tea production. At present, the cultivation of drought-resistant tea varieties by traditional methods is not only long in a cycle, low in efficiency, but also large in investment, which limits the cultivation speed of new varieties. Therefore, it is urgent to find a short cycle, high efficiency, and comprehensive evaluation method to speed up the selection of drought-resistant tea varieties.

Cultivating drought resistant tea variety is one of the effective ways to deal with drought stress. At present, the traditional method of cultivating drought resistant tea varieties not only has a long period, low efficiency, but also has a large investment, which limits the cultivation speed of new varieties. Therefore, it is urgent to find a method with short cycle, high efficiency and comprehensive evaluation to speed up the selection of drought resistant tea germplasm resources and the breeding of new drought resistant tea varieties. In order to evaluate the drought tolerance of tea germplasm resources, our research team used three machine learning models (Sizhou et al., 2021), SVM, RF and PLS, to model malondialdehyde (MDA), electrolyte leakage (EL), maximum efficiency of light system (*Fv/Fm*), soluble sugar (SS) and drought damage degree (DDD) of tea. The results showed that the CARS-PLS model of MDA was the best among the four physiological and biochemical indicators (R_cal_=0.96, R_p_=0.92, RPD=3.51). UVE-SVM model performs best in DDD (R_cal_=0.97, R_p_=0.95, RPD=4.28). Therefore, by using hyperspectral imaging technology to establish a machine learning model, the drought degree of tea seedlings under drought stress can be monitored. This method is not only fast and accurate, but also nondestructive.

In the past few years, many studies have recorded and explained the physiological and biochemical reactions of plants under drought conditions. Under drought stress, the content of soluble sugar in tea will slowly increase, making osmotic pressure increase, so as to improve the water holding capacity of cells (Palta et al., 2012). Drought stress will cause tea to produce too many active oxygen species and their derivatives (Impa et al., 2012), such as malondialdehyde. The increase of its content will lead to changes in cell membrane permeability, leading to plant senescence and death. The growth of tea plants is slow and often stops. The quality indicators of tea plants, such as the synthesis of tea polyphenols, will be affected, and their contents will gradually decrease (Upadhyaya et al., 2012). In the process of rehydration, the physiological activity of plants will tend to be normal or even better. Our research team’s previous research results show that MDA and SS can largely reflect the degree of stress on tea plants, and are positively correlated with the degree of stress. In this experiment, the factor of tea tree quality stability under drought stress was added. Because tea tree contains high content of polyphenols, its content changes with the change of stress degree. After comparing several indicators, it is believed that polyphenols are representative components of quality, and its content is negatively correlated with the degree of stress. This phenomenon has also been confirmed in the results of this experiment. Therefore, we choose the MDA, SS and total polyphenol as indicators to evaluate the drought tolerance of tea plants.

In this study, hyperspectral imaging technology was used to comprehensively evaluate the drought tolerance of different tea germplasm resources, including drought tolerance, post-stress recovery, and quality maintenance. CRITICAL method is based on the contrast strength of evaluation indicators and the conflict between indicators to comprehensively measure the objective weight of indicators, so we used the method to analyze the information expression of different types of physiological and biochemical data in the overall performance, and the three indexes of malondialdehyde, soluble sugar, and total polyphenol contents were weighted respectively to calculate the drought tolerance coefficient (DTC) of tea plants. MSC, SNV, 1D, 2D, S-G and other methods were used to preprocess hyperspectral data, and multiple feature band filtering algorithms such as UVE, CARS and SPA were used to extract feature bands. SVM, PLSR, RF and other modeling algorithms were used to model the characteristic band and stress tolerance index, and used to screen tea drought resistant germplasm resources.

## 2 Materials and methods

### 2.1 Experimental design

**Figure 1:**
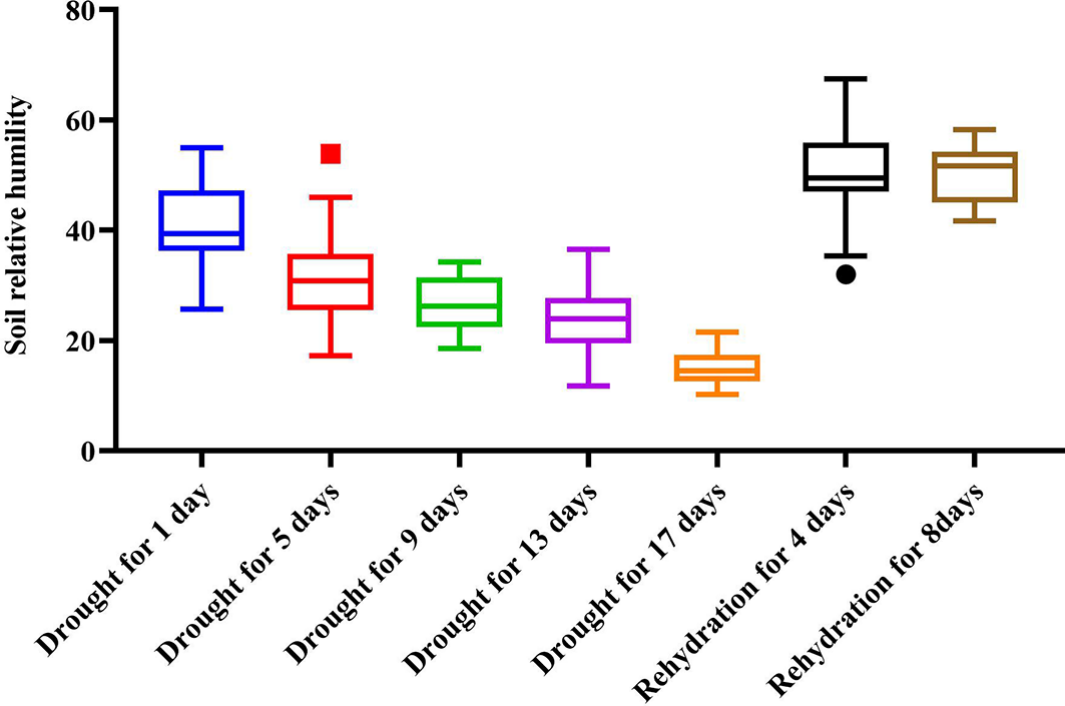
Value and trend of soil relative water content during the stress test.

The experiment was conducted at the scientific research greenhouse of Qingdao Agricultural University. The temperature of the greenhouse was 30° C during the day, the lighting time was 12 hours, the average light intensity was 10.6klus, and the temperature at night was 24° C, without light. There were ten tea varieties, including *SCZ, ZC 108, MS 9, QN 1, QN 21, QN 36, QN 38, JGY, JX*, and *XY 10*. The age of seedlings is three-year-old. There are 28 plants for each variety, with a total of 280 experimental seedlings. The test soil is mixed (40% subsoil, 40% matrix soil, 10% vermiculite, and 10% perlite). Tea seedlings were sterilized and planted in pots. The pre-culture stage was from November 24, 2021, to December 8, 2021, during which the soil moisture was maintained at 60% ~ 80%, the air humidity was maintained at 50%, and the greenhouse was ventilated for 2 ~ 4 hours every day. After the pre-culture stage, to simulate the natural water loss of tea plants under drought conditions, all water supply was stopped, and other conditions remained unchanged. The sampling started on December 9, 2021, with a sampling interval of 3 days. The sampling time was from 10:00 to 14:00 during the day when the physiological activity of tea plants is relatively significant. Four canopy samples were taken for each variety, with a total of 40 samples (Wei et al., 2009; Cao et al., 2015). The samplings were repeated five times during the drought stress. On December 28, 2021, the drought stress test was stopped and the rehydration test was started. The culture conditions were the same as those in the pre-culture stage, and the sampling interval was 3 days. Four canopy samples were collected for each variety, with a total of 40 samples. These samplings were repeated twice in the rehydration stage, and the test deadline was January 7, 2022. Two hundred and eighty samples were collected in this test. We used PMS710 soil moisture meter to measure and record the sample soil moisture during the test. **Figure 1** shows the average soil relative humidity of the sample plants measured at each sampling.

### 2.2 Data acquisition

#### 2.2.1 Acquisition and correction of hyperspectral data

**Figure 2:**
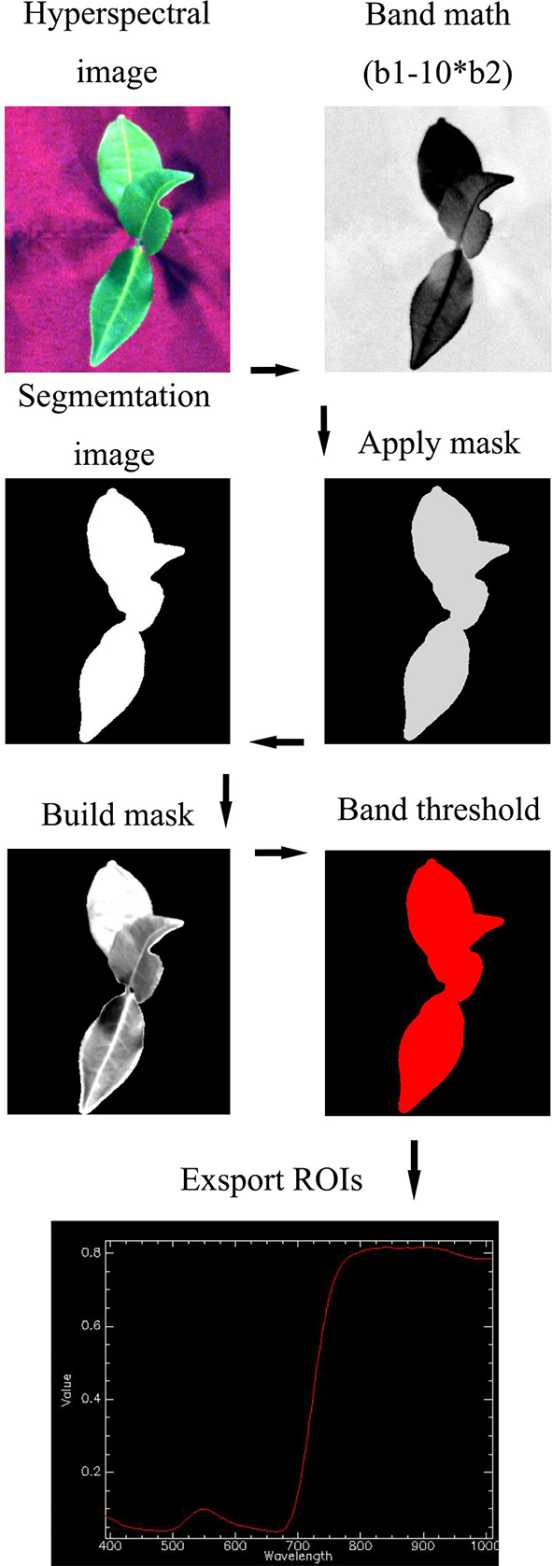
Flowchart of spectral data extraction.

Hyperspectral image acquisition system equipment mainly includes GaiaField Pro-V10 HSI camera (Jiangsu Dualix Imaging Technology Co., Ltd, China), Light source (color temperature light source is 3000 K, Hsia-ls-t-200w, China), supporting computer, darkroom and other components. Camera parameter settings: the exposure time is 22ms, the built-in lens pushing speed is 15s/cube, the spectral scanning range is 400 ~ 1000nm, the spectral interval is 1.667nm, the number of scanning bands is 360 bands, the image spatial resolution is 960 * 1040 (2X), the collected data size is 960 * 1101 * 360, the camera field of view angle is 22°, and the maximum DN value is 65552. Use the above equipment to collect the hyperspectral images of the tea tree canopy. The object distance is 20cm. Before and after the shooting, take the whiteboard and the black background respectively for later calibration. Specview (Jiangsu Dualix Imaging Technology Co., Ltd, China) is used to process and correct the original hyperspectral images sampled each time to obtain accurate hyperspectral image reflectance (between 0 and 1). In the software envi5.3 (Research Systems Inc., Boulder Co., United States), the mask method is used to extract the average spectral data of each hyperspectral image (**Figure 2**), and a 280 * 360 spectral matrix is obtained to facilitate subsequent processing. Leaf samples taken by hyperspectral camera will be used as samples for physiological and biochemical tests.

#### 2.2.2 Acquisition and analysis of physiological and biochemical indexes

To obtain more accurate data, the physiological and biochemical indexes of the samples were measured immediately after the hyperspectral images of the top view of the samples were collected. During the determination, each sample has 3 technical replicates, and the average value is taken as the test measurement value.

Determination of malondialdehyde content: the malondialdehyde content of the leaves corresponding to the hyperspectral images was determined using the malondialdehyde content Kit (Suzhou Grace Biotechnology Co., Ltd, Suzhou, China).

Determination of soluble sugar content: the soluble sugar content of the leaves corresponding to the hyperspectral images was determined by using the soluble sugar content Kit (Suzhou grace Biotechnology Co., Ltd, Suzhou, China).

Determination of total phenol: the total phenol content of the whole canopy was determined using the total phenol method Kit (Suzhou grace Biotechnology Co., Ltd, Suzhou, China). The content data of physiological and biochemical components of tea leaves measured with the kit are shown in **supplementary Table 1**

#### 2.2.3 Acquisition of DTC index of tea germplasm resources

To more intuitively understand the performance of different tea varieties at different stages, after obtaining the above physiological and biochemical data, the Tukey HSD method in SPSS software was used to analyze the significance of the data (P< 0.05), and different indicators were used to evaluate the difference of tea germplasm resources under water stress. The contribution degree of three indicators in drought evaluation is analyzed by using the CRITICAL objective weighting method, and the information amount and weight of different indicators were compared and obtained a comprehensive indicator DTC (Drought Tolerance Coefficient) which can comprehensively evaluate the drought tolerance, recovery ability and quality maintenance ability of tea plants.

### 2.3 Preprocessing of spectral data

To eliminate the influence of surface scattering, different scattering levels, and optical path changes on the diffuse reflection spectrum, improve the signal-to-noise ratio of spectral data, eliminate the baseline shift of spectral data caused by environmental interference, and diffuse reflection and spectrum overlap, we used standard normal variable transformation (SNV) (Sandak et al., 2016), multivariate scattering correction (MSC) (Zhao et al., 2005; Shao et al., 2012), S-G smoothing (Lu et al., 2019)(Savitzky-Golay). The first-order differential (Tian et al., 2005) (1D) and the second-order (Chu, 2004) differential (2D) preprocess the extracted spectral data in different combinations. The relevant formula is as follows:


$$S\tan dard\;normal\;transformation:X_{SNV}=\frac{x-\bar{x}}{\sqrt{\frac{\sum_{k=1}^{m}{\left(x_{k}-\bar{x}\right)}^{2}}{\left(m-1\right)}}}$$

Where $\bar{x}=\frac{\sum_{k=1}^{m}x_{k}}{m}$ , m is the number of wavelength points, k = 1, 2, …, m


$$Multiple\;scattering\;correction:\bar{X(i)}=\frac{\sum_{i=1}^{n}x(i)}{n}$$

$$X(i)=m(i)*\bar{x(i)}+b(i)$$

$$X{\left(i\right)}_{\left(msc\right)}=\frac{x(i)-b(i)}{m(i)}$$

Where *X* is the original spectral matrix of the sample, *X*(i), *m*(i), *b*(i), *X*(i)_(_ *_msc_*_)_is the average value of the surface original spectrum, the regression constant, the regression coefficient, and the MSC correction spectrum of the i the sample.


$$S-G\;smoothing:X_{i}^{*}=\frac{\sum_{j=-r}X_{i}+W_{j}}{\sum_{j=-r}^{r}W_{j}}$$

Where $X_{i}^{*}$ , *X_i_* is the spectral data point before and after S-G smoothing, *W_j_* is a weight factor obtained by smoothly moving the window using the window width 2R + 1.


$$First\;derivative:\frac{{\text{d}}_{y}}{{\text{d}}_{\lambda }}=\frac{y_{i+1}-y_{i}}{\Delta \lambda }$$

$$Second\;derivative:\frac{{\text{d}}_{y}^{2}}{{\text{d}}_{\lambda^{2}}}=\frac{y_{i+1}-2y_{i}+y_{i-1}}{\Delta \lambda^{2}}$$

### 2.4 Selection of characteristic wavelength

To improve the operational efficiency and accuracy of the model, we used three algorithms, namely continuous projection algorithm (SPA), competitive adaptive reweighted sampling (CARS), and uninformative variable elimination (UVE), to screen the whole bands (Chen and Chen, 2005; Wu et al., 2009; Shi et al., 2018), and obtain the characteristic bands with the strongest correlation with the dependent variable as the input of the model.

### 2.5 Model establishment and evaluation

In this study, a total of 280 data sets of tea canopy were collected, and each data set was composed of average hyperspectral data and DTC. The data set was divided into training sets (210) and validation sets (70) according to the ratio of 3:1. After the spectral data were processed in the above process, the corresponding prediction models were established by using support vector machine (SVM), random forest (RF) and partial least squares regression (PLSR) (Vapnik, 1998; Carrascal et al., 2010; Dong and Huang, 2013; Li, 2013; Zhou, 2016). The stability and accuracy of the model were evaluated by the determination coefficient (R^2^), root means square error (RMSE), and mean absolute percentage error (MAPE) (Dodge, 2006; Aptula et al., 2010; Alam Akbar and Subiakto, 2013). R^2^, RMSE, and MAPE are calculated as follows:


$$R^{2}=\frac{\sum_{i=1}^{n}{\left({\hat{y}}_{i}-{\bar{y}}_{i}\right)}^{2}}{\sum_{i=1}^{n}{\left(y_{i}-{\bar{y}}_{i}\right)}^{2}}$$

$$RMSE=\sqrt{\frac{1}{n}\sum_{i=1}^{n}{\left({\hat{y}}_{i}-y_{i}\right)}^{2}}$$

$$MAPE=\frac{100\%}{n}\sum_{i=1}^{n}\left|\frac{{\hat{y}}_{i}-y_{i}}{y_{i}}\right|$$

Where *n* is the number of samples, *y_i_* is the true value of the sample target variable, ${\hat{y}}_{i}$ is the target variable value predicted using the regression model.

## 3 Results and analysis

### 3.1 Comprehensive analysis of tea germplasm resources with drought tolerance and establishment of DTC

**Figure 3:**
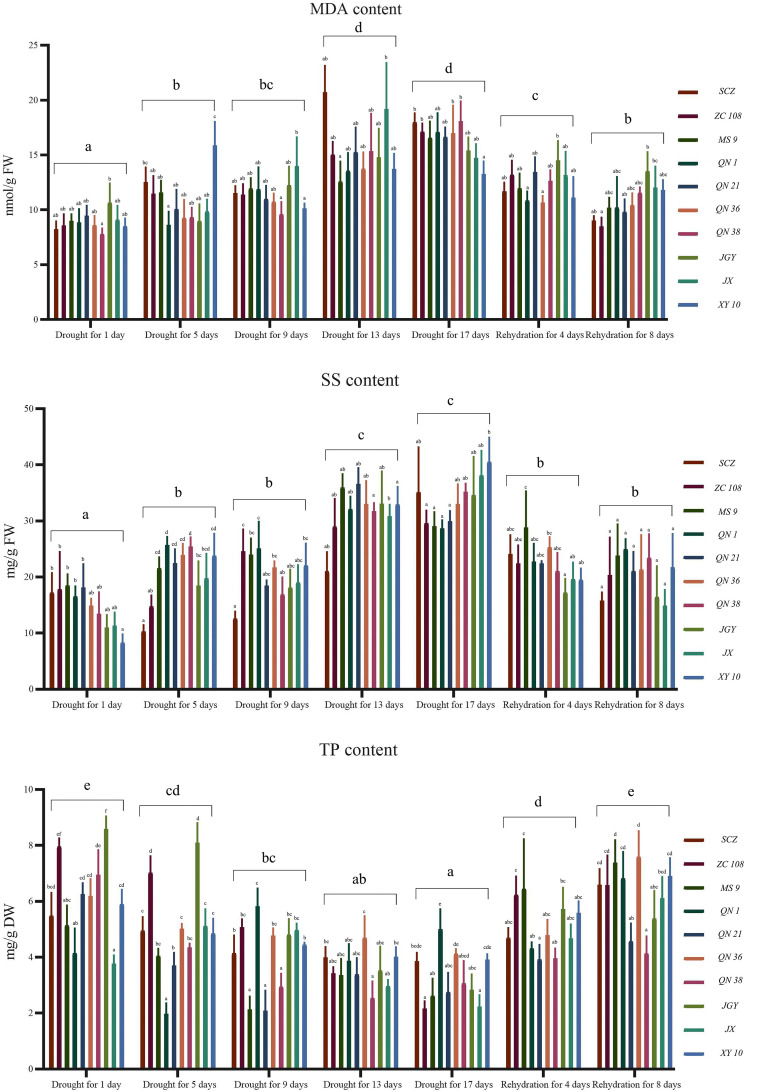
Statistical analysis of physiological and biochemical indexes (malondialdehyde, soluble sugar and total polyphenol content) data of tea germplasm resources in different periods. MDA, malondialdehyde; SS, soluble sugar; TP, total phenol.

**Figure 4:**
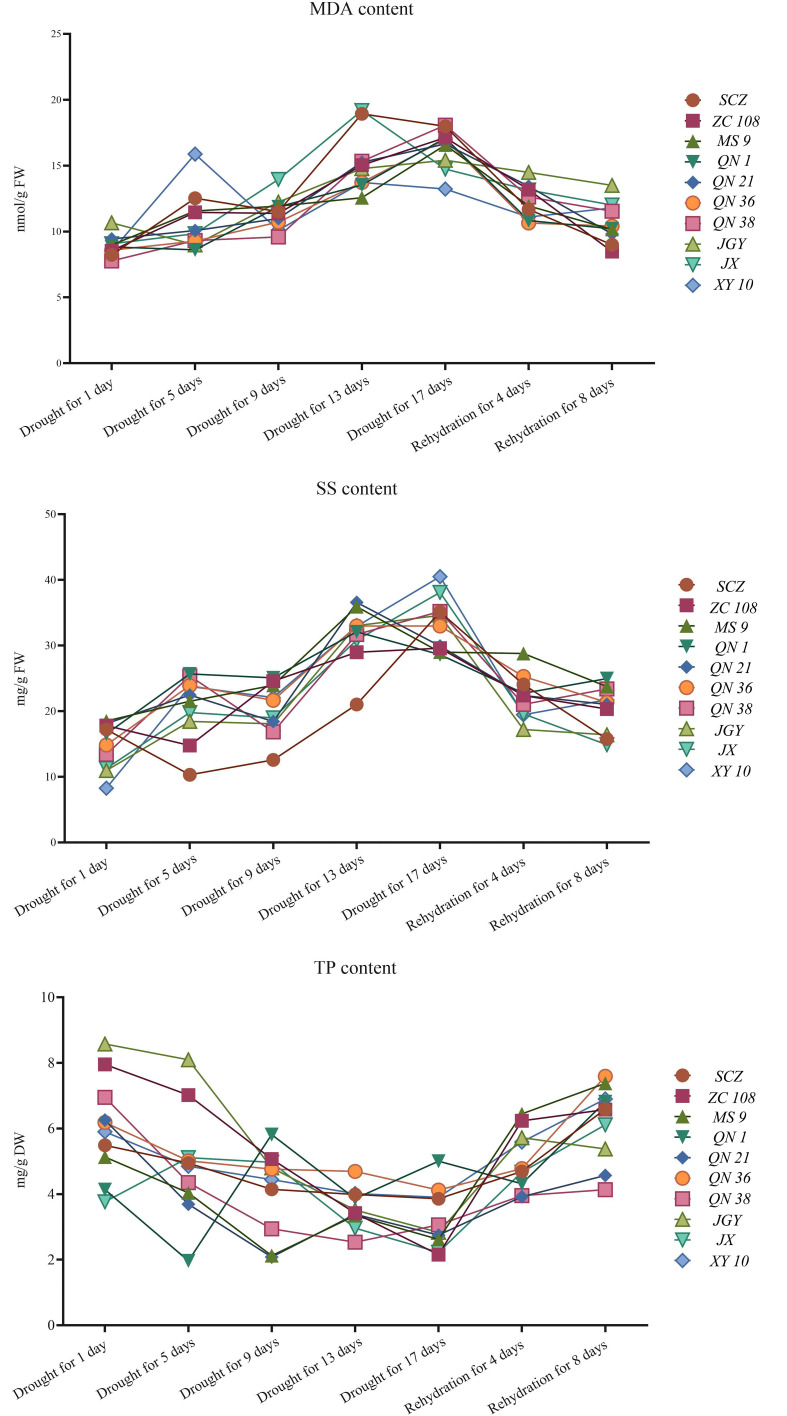
Change trend of physiological indexes (malondialdehyde, soluble sugar and tea polyphenol content) of tea germplasm resources.

It can be seen from **Figure 3** that the index difference among different treatments is high, in which the content of malondialdehyde and soluble sugar was positively correlated with the stress time, and the content of total phenol is negatively correlated. The physiological indexes of different varieties of tea plants changed during drought stress. The contents of malondialdehyde and soluble sugar of all varieties increased first and then decreased, and reached a peak from 13 to 17 days of drought. Among them, the MDA content of *‘JX’* reached the highest value of 19.18nmol/g on the 13th day of drought, and the soluble sugar of *‘XY 10’* reached the highest value of 40.15mg/g on the 17th day of drought. The content of total phenol decreased first and then increased, and reached the lowest level on the 17th day of the drought. Among them, the content of *‘ZC 108’* was the lowest, 2.15mg/g. Under the same stress treatment, the difference in component content among varieties decreased with the increase of stress time. On the first day of drought stress, the highest MDA content of *‘JGY’* was 37.06% higher than that of *‘QN 38’*. On the 17th day of drought stress, the highest MDA content of *‘QN 38’* was 36.68% higher than that of Xinyang 10. On the first day of drought stress, the highest SS content of *‘MS 9’* was 56.94% higher than that of Xinyang 10. On the 17th day of drought stress, the highest SS content of *‘XY 10’* was 29.32% higher than that of *‘QN 1’*, with a relative decrease of 17.62%. On the first day of drought stress, the highest TP content of *‘JGY’* was 56.12% higher than that of *‘JX’*. On the 17th day of drought stress, the highest TP content of *‘QN* 1’ was 55.69% higher than that of *‘JX’*. To better understand the relationship between drought tolerance and water stress of different varieties, we statistically analyzed the content changes of physiological and biochemical indexes of tea varieties in different periods. The fluctuation range of indexes is shown in **Figure 4**, and the descriptive statistical results are shown in **Table 1**.

**Table 1:** Changes of physiological and biochemical data of tea germplasm resources in different periods.

Index	Varieties	Drought for 1 day	Drought for 5 days	Drought for 9 days	Drought for 13 days	Drought for 17 days	Rehydration for 4 days	Rehydration for 8 days
MDA	*‘SCZ’*	8.23 ± 0.36	12.52 ± 0.66	11.51 ± 0.32	18.93 ± 1.28	17.98 ± 0.41	11.69 ± 0.39	8.99 ± 0.22
	*‘ZC 108’*	8.54 ± 0.52	11.45 ± 0.81	11.38 ± 0.48	15.03 ± 0.58	17.13 ± 0.37	13.18 ± 0.65	8.48 ± 0.39
	*‘MS 9’*	8.97 ± 0.31	11.56 ± 0.54	11.92 ± 0.49	12.56 ± 0.92	16.56 ± 0.74	11.94 ± 0.67	10.16 ± 1.47
	*‘QN 1’*	8.83 ± 0.62	8.61 ± 0.61	11.87 ± 0.99	13.53 ± 0.82	17.09 ± 0.86	10.83 ± 0.42	10.21 ± 1.39
	*‘QN 21’*	9.44 ± 0.47	10.07 ± 0.87	10.98 ± 0.60	15.25 ± 1.12	16.64 ± 0.43	13.43 ± 0.68	9.82 ± 0.57
	*‘QN 36’*	8.58 ± 0.43	9.26 ± 0.82	10.72 ± 0.38	13.72 ± 0.75	16.97 ± 1.26	10.65 ± 0.31	10.41 ± 0.55
	*‘QN 38’*	7.77 ± 0.27	9.31 ± 0.44	9.59 ± 0.56	15.33 ± 1.71	18.07 ± 0.89	12.62 ± 0.49	11.54 ± 0.25
	*‘JGY’*	10.65 ± 0.87	8.98 ± 0.76	12.24 ± 0.85	14.77 ± 1.31	15.40 ± 0.59	14.49 ± 0.89	13.51 ± 0.88
	*‘JX’*	9.07 ± 0.64	9.83 ± 0.55	13.95 ± 1.33	19.18 ± 2.10	14.74 ± 0.63	13.14 ± 1.07	12.01 ± 0.96
	*‘XY 10’*	8.50 ± 0.35	15.87 ± 1.07	10.11 ± 0.23	13.72 ± 0.70	13.22 ± 0.59	11.83 ± 0.85	11.79 ± 0.46
SS	*‘SCZ’*	17.21 ± 1.74	10.32 ± 0.56	12.58 ± 0.63	21.02 ± 1.71	35.12 ± 3.99	24.07 ± 1.68	15.76 ± 0.75
	*‘ZC 108’*	17.81 ± 3.33	14.77 ± 0.97	24.59 ± 1.94	28.99 ± 2.47	29.60 ± 1.12	22.38 ± 1.63	20.34 ± 3.35
	*‘MS 9’*	18.51 ± 0.99	21.534 ± 0.99	23.95 ± 1.46	35.93 ± 1.19	29.01 ± 1.28	28.79 ± 3.24	23.76 ± 2.81
	*‘QN 1’*	16.54 ± 0.89	25.68 ± 0.74	25.07 ± 2.39	32.11 ± 1.09	28.63 ± 0.75	22.73 ± 1.59	24.97 ± 0.89
	*‘QN 21’*	18.11 ± 2.09	22.47 ± 1.24	18.43 ± 0.48	36.58 ± 1.42	29.92 ± 0.92	22.38 ± 0.22	21.04 ± 1.71
	*‘QN 36’*	14.85 ± 0.64	23.91 ± 1.01	21.66 ± 0.57	32.99 ± 2.06	32.99 ± 1.77	25.29 ± 0.91	21.36 ± 3.05
	*‘QN 38’*	13.44 ± 1.92	25.38 ± 0.84	16.86 ± 1.54	31.70 ± 0.74	35.20 ± 0.72	21.04 ± 1.63	23.39 ± 2.11
	*‘JGY’*	10.96 ± 1.13	18.45 ± 2.18	18.08 ± 1.61	33.04 ± 2.89	34.61 ± 3.39	17.21 ± 1.24	16.41 ± 2.77
	*‘JX’*	11.28 ± 1.19	19.81 ± 2.16	18.98 ± 1.57	30.84 ± 1.03	38.11 ± 2.19	19.58 ± 1.49	14.91 ± 1.39
	*‘XY 10’*	8.27 ± 0.76	23.784 ± 1.96	22.08 ± 1.93	32.94 ± 1.56	40.51 ± 2.17	19.47 ± 1.01	21.74 ± 2.97
TP	*‘SCZ’*	5.48 ± 0.41	4.94 ± 0.25	4.14 ± 0.32	3.99 ± 0.19	3.86 ± 0.15	4.69 ± 0.18	6.59 ± 0.28
	*‘ZC 108’*	7.950.15	7.02 ± 0.29	5.07 ± 0.14	3.42 ± 0.11	2.15 ± 0.13	6.23 ± 0.33	6.58 ± 0.53
	*‘MS 9’*	5.13 ± 0.36	4.04 ± 0.13	2.12 ± 0.23	3.34 ± 0.29	2.62 ± 0.31	6.44 ± 0.89	7.37 ± 0.41
	*‘QN 1’*	4.13 ± 0.44	1.96 ± 0.19	5.81 ± 0.32	3.86 ± 0.29	5.01 ± 0.36	4.31 ± 0.11	6.81 ± 0.47
	*‘QN 21’*	6.25 ± 0.19	3.69 ± 0.23	2.08 ± 0.36	3.38 ± 0.29	2.75 ± 0.34	3.92 ± 0.26	4.57 ± 0.32
	*‘QN 36’*	6.18 ± 0.31	5.01 ± 0.09	4.76 ± 0.13	4.69 ± 0.39	4.12 ± 0.09	4.78 ± 0.28	7.59 ± 0.46
	*‘QN 38’*	6.95 ± 0.44	4.35 ± 0.07	2.94 ± 0.23	2.53 ± 0.29	3.06 ± 0.39	3.95 ± 0.18	4.13 ± 0.31
	*‘JGY’*	8.57 ± 0.23	8.09 ± 0.35	4.79 ± 0.28	3.51 ± 0.43	2.83 ± 0.27	5.72 ± 0.38	5.37 ± 0.49
	*‘JX’*	3.76 ± 0.15	5.11 ± 0.29	4.97 ± 0.11	2.96 ± 0.11	2.22 ± 0.21	4.67 ± 0.25	6.11 ± 0.38
	*‘XY 10’*	5.91 ± 0.26	4.85 ± 0.26	4.44 ± 0.04	4.01 ± 0.17	3.91 ± 0.09	5.59 ± 0.21	6.89 ± 0.32

The data in the table are the mean of three repetitions ± standard error of mean.

Through the analysis of the change trend chart and descriptive data of malondialdehyde content, it is known that the average value and dispersion coefficient of *‘MS 9’*, *‘QN 1’*, *‘QN 36’*, *‘QN 38’*, and *‘XY 10’* are low, and the oxidative metabolic activity is low during the water stress period, and the comprehensive performance is good. Through the analysis of the change trend chart of soluble sugar content and descriptive data, it is known that the average value and dispersion coefficient of *‘ZC 108’*, *‘MS 9’*, *‘QN 1’*, *‘QN 21’*, and *‘QN 36’* were low, the osmotic pressure was maintained well, and the comprehensive performance is good. According to the analysis of the change trend chart of total phenol content and descriptive data, the total phenol content of *‘ZC 108’*, *‘QN 1’*, *‘QN 36’*, *‘JX’*, and *‘XY 10’* remained at a high level and the dispersion coefficient was small, and the quality retention ability was strong during the stress period. However, the above assessments are all identification of single indicators and do not meet the conditions for comprehensive assessment and identification (Liang et al., 2014).

**Figure 5:**
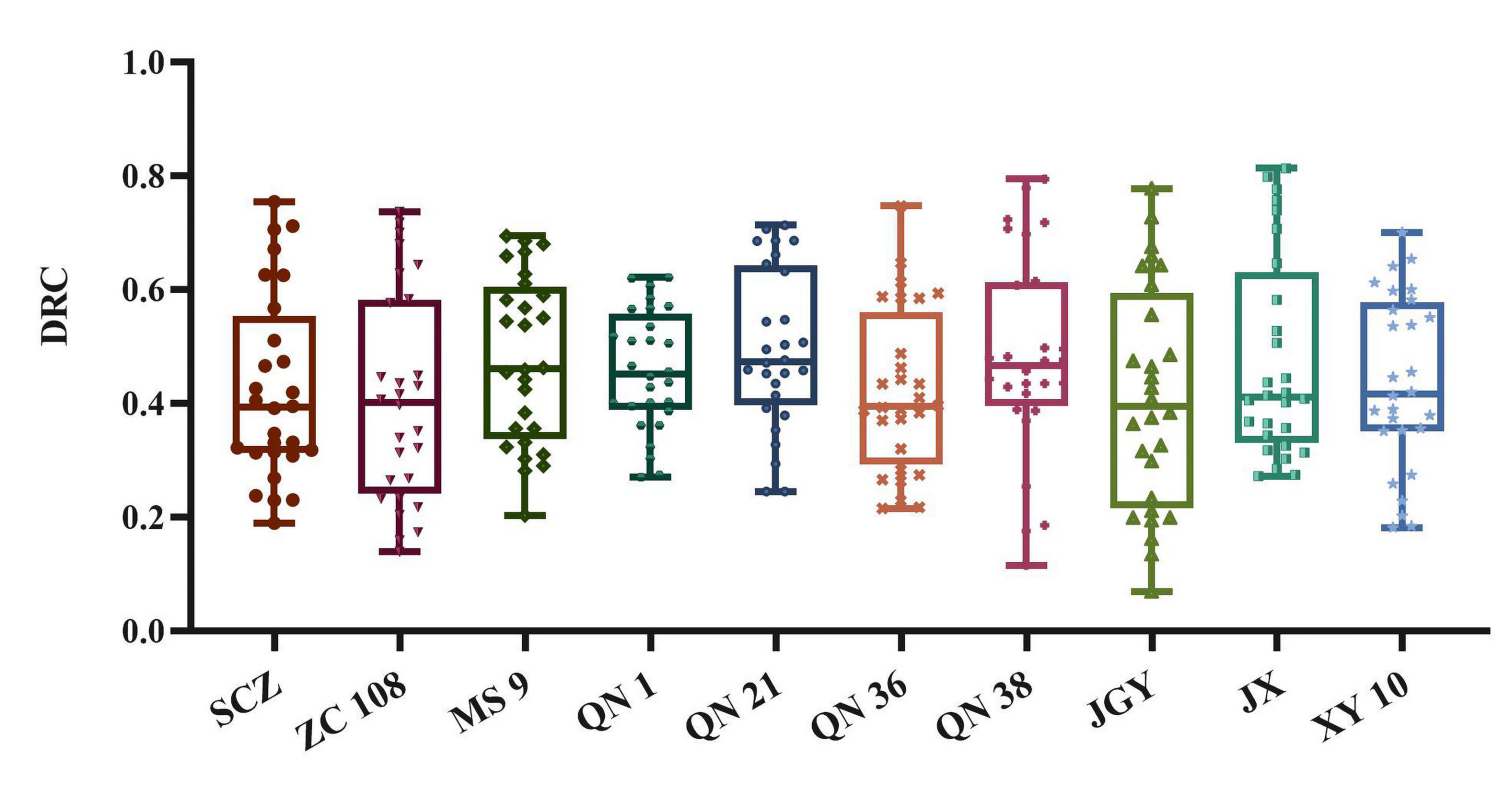
Statistical analysis results and distribution of DTC of different tea germplasm resources.

To further more comprehensively and directly identify the tea germplasm resources with drought tolerance, we used the CRITICAL objective weighting method to analyze the content of malondialdehyde, soluble sugar, and total polyphenols. It was found that the information contents of MDA, SS, and TP were 0.21, 0.209, and 0.227 respectively, accounting for 32.57%, 32.32%, and 35.11% respectively. We used the range method to make the data of physiological and biochemical indexes positively correlated with stress time. After weighted calculation for all individuals, we obtained the DTC index of each tea individual. The higher the DTC index, the stronger the drought tolerance ability, the lower the DTC index, and the weaker the drought tolerance ability. The DTC distribution of each variety is shown in **Figure 5**.

It can be seen from **Figure 5** that in the distribution with small dispersion, *‘QN1’*, *‘QN 21’*, and *‘MS 9’* account for a high proportion. As shown in **Table 2**, by comparing the percentages of different varieties in the overall median and the overall average, the comprehensive drought tolerance of all varieties is ranked. The ranking results from high to low are: *QN 36*, *SCZ*, *ZC 108*, *JX*, *JGY*, *XY 10*, *QN 1*, *MS 9*, *QN 38* and *QN 21*. Among them, *‘QN 38’* and *‘QN 21’* had good drought tolerance, but their quality stability is poor. The reason may be that the polyphenol content of these two varieties is lower than that of other varieties during drought stress or water sufficiency period, so the comprehensive score is low.

**Table 2:** Proportion of DTC of different tea germplasm resources in all tested individuals.

	Number of samples less than the overall DTC average (0.4487)	Percentage (%)	Number of samples less than the overall DTC median (0.4344)	Percentage (%)
*‘SCZ’*	18	6.43%	18	6.43%
*‘ZC 108’*	19	6.79%	17	6.07%
*‘MS 9’*	12	4.29%	11	3.93%
*‘QN 1’*	14	5%	12	4.29%
*‘QN 21’*	9	3.21%	8	2.86%
*‘QN 36’*	19	6.79%	18	6.43%
*‘QN 38’*	13	4.64%	9	3.21%
*‘JGY’*	17	6.07%	16	5.71%
*‘JX’*	18	6.43%	16	5.71%
*‘XY 10’*	16	5.71%	15	5.36%

**Figure 6:**
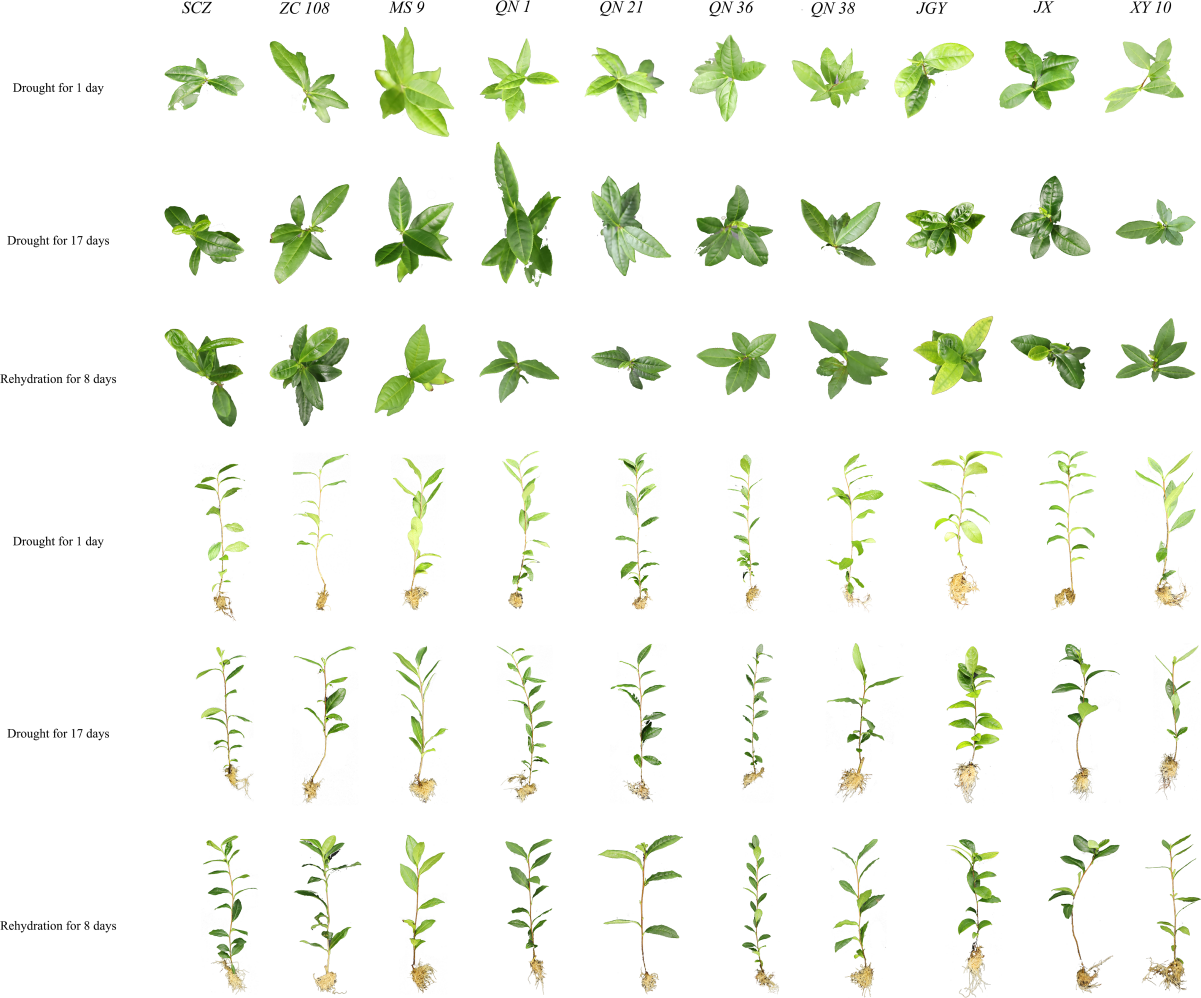
Phenotypic changes of ten tea germplasm resources in three experimental stages (drought for 1day, drought for17days and rehydration for 8days).

In the traditional methods, the destructive detection is time-consuming and laborious, and the manual observation of tea seedlings has a certain delay error and subjective error. Therefore, we recorded not only the spectral data, but also the phenotypic change data of the samples during the test to ensure that there was no obvious change in the aboveground part of the samples during the test. As shown in **Figure 6**, in this experiment, there was little difference in the phenotype of the aboveground tissues of various tea varieties before and after stress, at the end of stress, and the end of rehydration. On the contrary, the root system of the underground part of tea seedlings developed and grew. Therefore, it is difficult to select varieties and individuals with both drought tolerance and quality maintenance ability by observing the difference in the aboveground part of tea germplasm resources during drought stress. Hyperspectral imaging technology has changed the traditional methods of germplasm resource identification and can speed up the selection process of tea drought-resistant varieties in terms of time and efficiency.

### 3.2 Processing results of hyperspectral data

**Figure 7:**
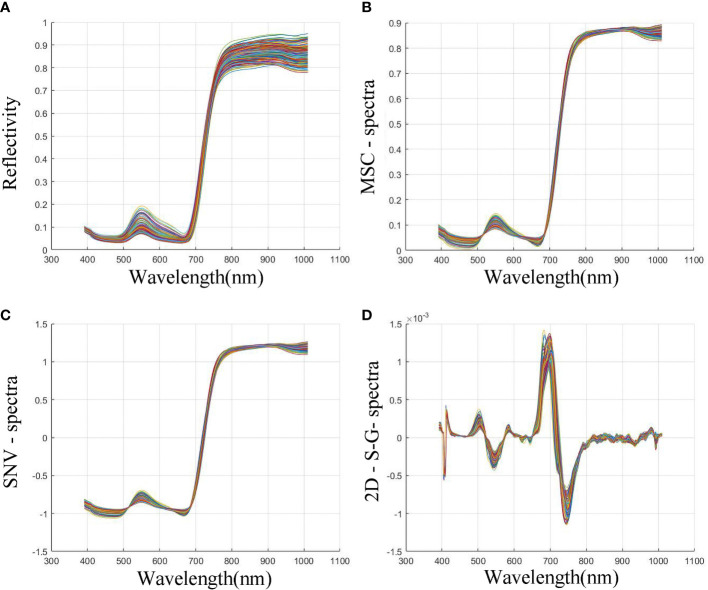
Changes of spectral data under different pretreatment methods. **(A)** Original bands image; **(B)** Bands image after MSC processing; **(C)** Bands image after SNV processing; **(D)** Bands image after second-order differential and S-G method processing.

To improve the reliability of spectral data, the preprocessing visualization data of average spectral data of all samples are shown in **Figure 7**. Compared with the original data, the spectral data after MSC correction enhances the correlation between the spectral data. SNV expand the upper and lower limits of the data and eliminated the diffuse reflection of most of the data. To enhance the stability of the data and improve the signal-to-noise ratio, we subsequently used the optimal S-G smoothing and differentiation method to process the hyperspectral data. The data after the S-G smoothing and differentiation method were smoother in distribution and has convexity. The data visualization is shown in **Figure 7** (d). After the later evaluation of the model, we found that such processing is more conducive to the later feature filtering algorithm to extract feature bands.

**Figure 8:**
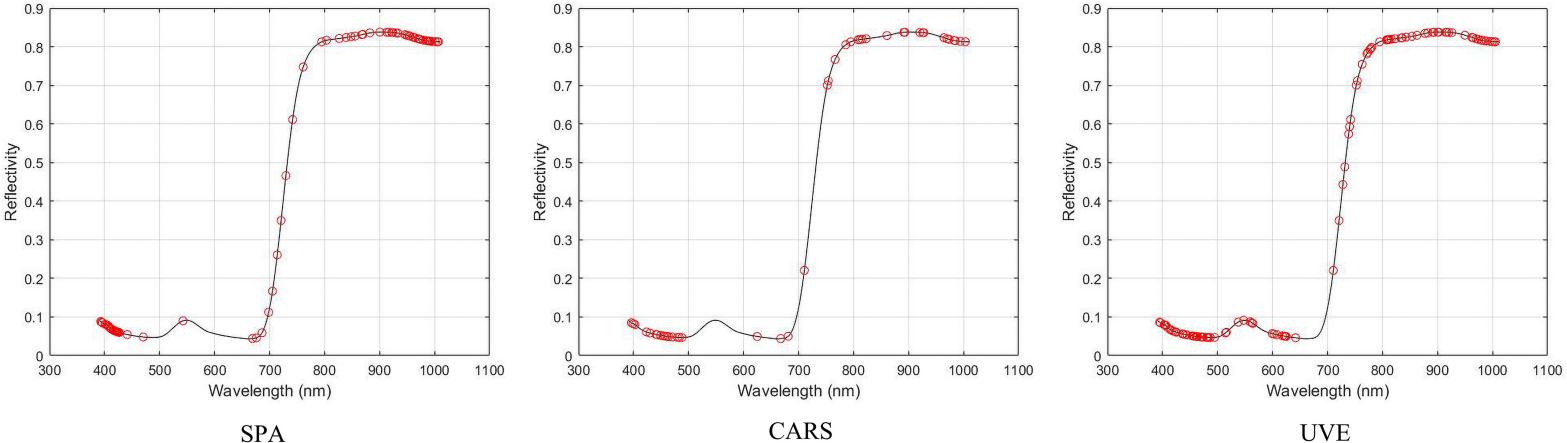
The characteristic bands screened by SPA, CARS and UVE algorithms.

For the preprocessed average spectral data and dependent variable data set, SPA, CARS, and UVE algorithms were used to screen the characteristic bands. The variable screening results of the three algorithms are shown in **Figure 8**. The optimal characteristic bands screened by SPA, CARS, and UVE algorithms are 95, 42, and 63 respectively. The characteristic bands screened by the SPA algorithm are sparsely distributed between 500 ~ 800nm, the characteristic bands screened by the CARS algorithm are sparsely distributed within 600 ~ 800, the characteristic bands screened by the UVE algorithm are distributed around 550nm and 600nm, and between 700 ~ 800nm. The characteristic bands screened by the three algorithms are mainly distributed between 391 ~ 440nm and 800 ~ 1000nm. This may be because, in the visible light range of 400-700nm (Wang et al., 2018), tea absorbs a large amount of visible light. However, under drought stress, tea photosynthesis weakens visible light reflection increases and the original spectral reflectance of the canopy increases. In the near-infrared range of 700-1000 nm, the changes in the internal structure of the leaves affected the spectral reflectance of the canopy (Mu et al., 2012; Xu et al., 2017). We will continue to study the relationship between this band interval and tea phenotype.

**Figure 9:**
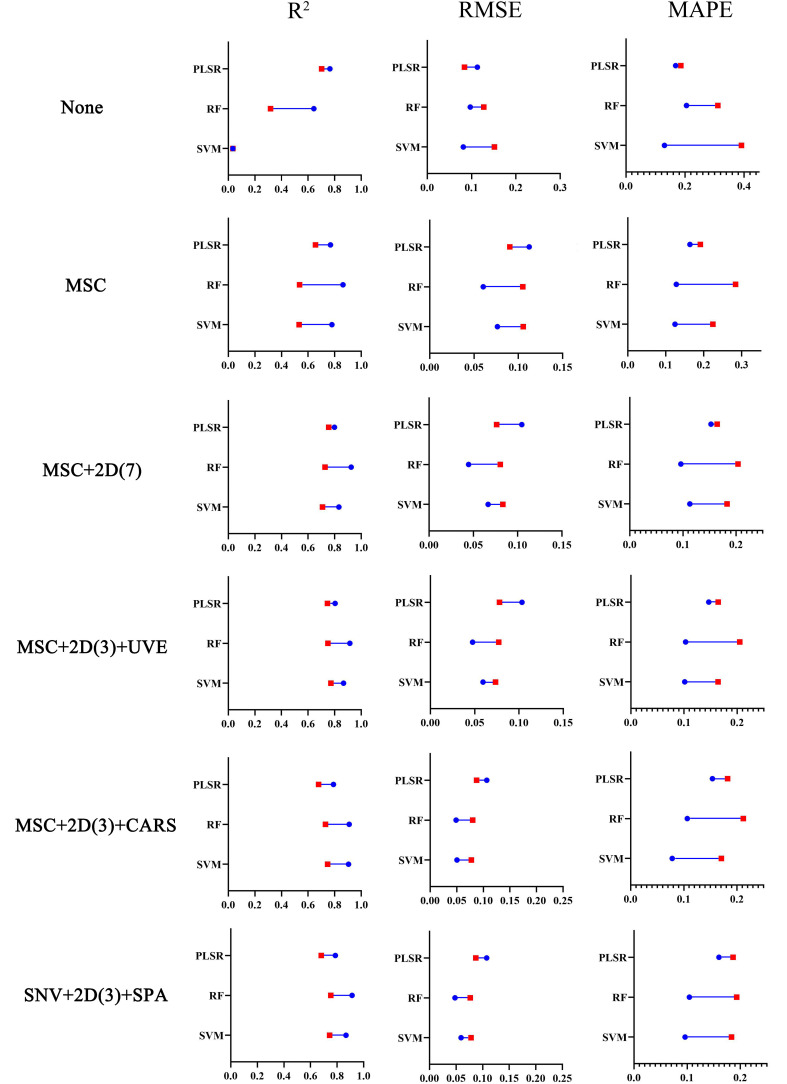
Visualization of evaluation index of all modeling methods.

The original band data set, the spectral data set with different preprocessing, and the optimal characteristic band data set were respectively input into SVM, RF, and PLSR algorithms. The model evaluation results of different treatment methods are shown in **Figure 9**. **Table 3** records more detailed model data. It can be seen from the scatter plot that the models established based on the original spectral data set have the worst effect, such as None-SVM, None-PLSR, and None-RF models, among them, the PLSR model ( $R_{te}^{2}=0.7$ , RMSE_te_ = 0.84, MAPE_te_ = 0.19) based on the original spectrum performs best and has a good prediction effect, but the prediction error is not meeting expectations. To further reduce the prediction error, we established a model based on scattering correction. But these models have poor prediction accuracy and a long calculation time, among them, the MSC-PLSR model ( $R_{te}^{2}=0.66$ , RMSE_te_ = 0.09, MAPE_te_ = 0.19) performs best. The prediction effect of this model performs quite ordinary, but the prediction error is small. To further improve the prediction effect and reduce the prediction error, we established a model based on scattering correction and mathematical transformation processing (1D, 2D, and S-G). Among them, the MSC-2D (5)-PLSR model ( $R_{te}^{2}=0.75$ , RMSE_te_ = 0.076, MAPE_te_ = 0.16) performs best. The prediction effect and prediction error of this model are excellent. To improve the operation speed of the model and improve the prediction accuracy of the model, we established a model based on scattering correction. Among the models of mathematical transformation processing and feature filtering algorithm, the MSC-2D (3)-UVE-SVM model ( $R_{te}^{2}=0.77$ , RMSE_te_ = 0.073, MAPE_te_ = 0.16) is the best. The prediction accuracy and prediction error of this model are the best among all models, which improves the accuracy of prediction and reduces the calculation time of model prediction. This shows that, the accuracy of the model established based on a variety of algorithms has been greatly improved, which may be because a variety of preprocessing algorithms have improved the signal-to-noise ratio of spectral data, increased the analysis and regression ability of linear and nonlinear data, and provided a more diversified calculation method for the model (Zhang et al., 2019). Through the comparison of all the above prediction models, it can be found that: the prediction accuracy of the PLSR model is moderate, and the prediction error is larger than that of the RF model and SVM model. The model established by the RF algorithm is mediocre in prediction accuracy and prediction error, and the SVM model is better than the former in prediction accuracy and prediction error.

**Table 3:** Evaluation results of different prediction models.

Pretreatment 1	Pretreatment 2	Pretreatment 3	Feature extraction algorithm	Modeling method	Training set			Testing set		
					R^2^	RMSE	MAPE	R^2^	RMSE	MAPE
None	None	None	None	SVM	0.034	0.081	13.04%	0.034	0.15	39.09%
None	None	None	None	RF	0.64	0.097	20.50%	0.32	0.13	31.12%
None	None	None	None	PLSR	0.76	0.11	16.82%	0.7	0.08	18.60%
MSC	None	None	None	SVM	0.78	0.076	12.45%	0.53	0.11	22.41%
SNV	None	None	None	RF	0.86	0.06	12.80%	0.54	0.11	28.38%
MSC	None	None	None	PLSR	0.76	0.11	16.37%	0.66	0.1	19.12%
MSC	2D	S-G (7)	None	SVM	0.83	0.067	11.30%	0.71	0.083	18.31%
SNV	2D	S-G (3)	None	RF	0.92	0.045	9.62%	0.73	0.08	20.36%
MSC	2D	S-G (5)	None	PLSR	0.8	0.1	15.28%	0.76	0.076	16.44%
MSC	2D	S-G (3)	UVE	SVM	0.9	0.05	7.83%	0.75	0.078	17.07%
MSC	2D	S-G (3)	UVE	RF	0.91	0.048	10.30%	0.75	0.077	20.49%
SNV	2	S-G (5)	UVE	PLSR	0.8	0.1	15.01%	0.74	0.079	16.15%
MSC	2	S-G (3)	CARS	SVM	0.9	0.05	7.83%	0.75	0.078	17.07%
MSC	2D	S-G (3)	CARS	RF	0.91	0.049	10.65%	0.73	0.08	21.21%
SNV	2D	S-G (11)	CARS	PLSR	0.79	0.11	15.38%	0.68	0.087	18.25%
SNV	2D	S-G (3)	SPA	SVM	0.87	0.059	9.62%	0.74	0.078	18.34%
SNV	1D	S-G (11)	SPA	RF	0.91	0.048	10.43%	0.75	0.077	19.32%
SNV	2D	S-G (5)	SPA	PLSR	0.8	0.1	15.28%	0.76	0.076	16.43%

**Figure 10:**
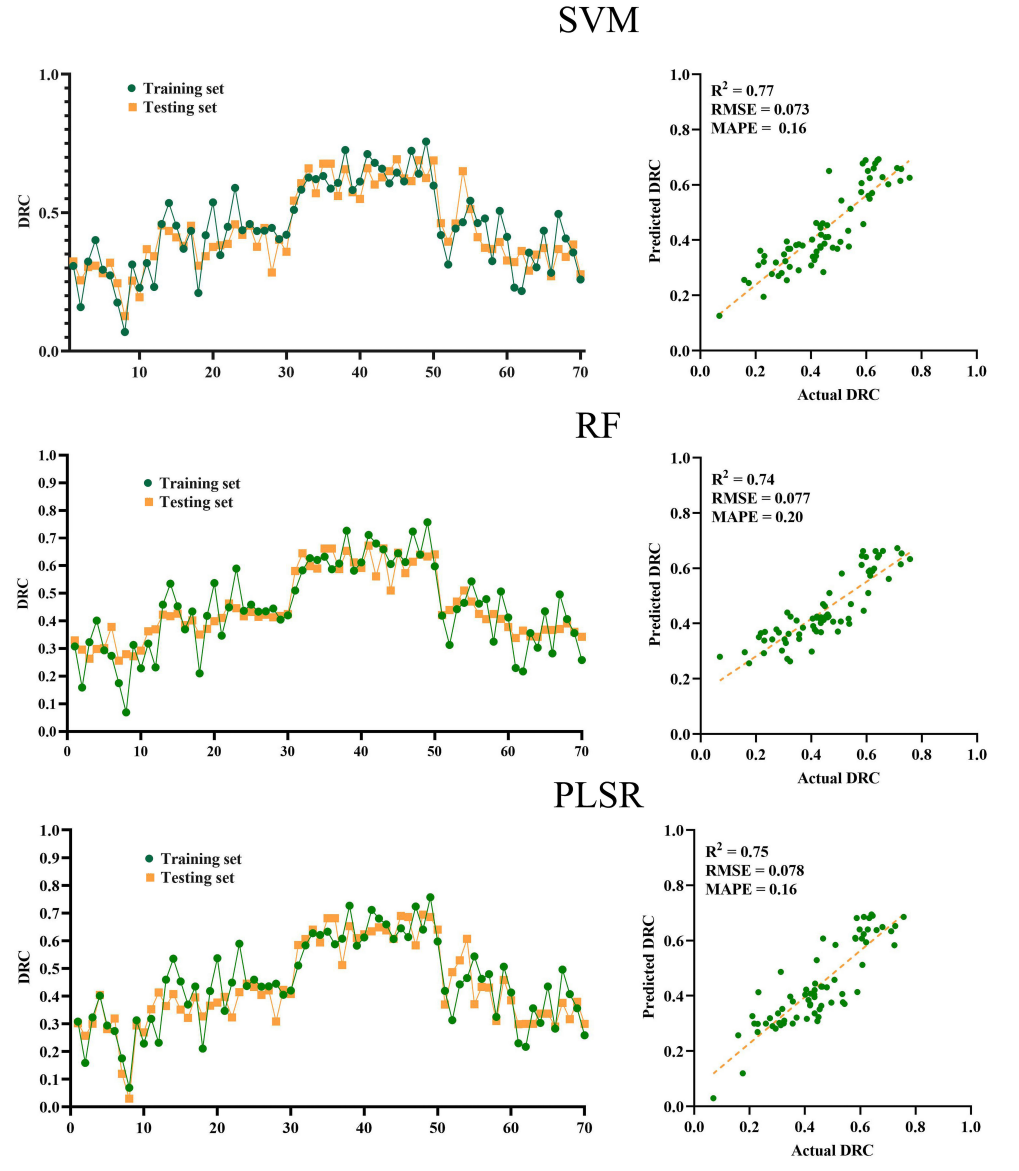
Modeling results and regression graphs of the three optimal algorithms (Top down are SVM, RF and PLSR).

After comparing the accuracy and error of all prediction models, we screened three models for horizontal comparison. These three models are MSC-2D (3)-UVE-SVM, MSC-2D (3)-UVE-RF and SNV-2D (5)-UVE-PLSR. **Figure 10** shows the prediction and regression diagrams of SVM, RF and PLSR respectively. According to the regression degree and prediction trend shown in **Figure 10**, it can be seen that in this experiment, the various indexes of this SVM model are slightly better than those of RF and PLSR models, so the optimal prediction model combination of drought-resistant tea germplasm DTC is MSC-2D (3)-UVE-SVM model ( $R_{te}^{2}=0.77$ , RMSE_te_ = 0.073, MAPE_te_ = 0.16).

## 4 Conclusion

In this experiment, drought and rehydration experiments were conducted on several tea germplasm resources, physiological, biochemical, and hyperspectral data were collected, the weights of different physiological and biochemical indexes in evaluating the drought tolerance of tea plants were analyzed, and the original spectral data were cut and processed by different algorithms, and the corresponding DTC prediction model was established, and the feasibility and advantages of this method were analyzed. The results of physiological and biochemical detection and analysis showed that the tea germplasm resources with drought tolerance from strong to weak were: *QN 36*, *SCZ*, *ZC 108*, *JX*, *JGY*, *XY 10*, *QN1*, *MS 9*, *QN 38*, and *QN 21*, and the best tea germplasm resource with drought tolerance model established in this experiment was MSC-2D (3)-UVE-SVM model ( $R_{te}^{2}=0.77$ , RMSE_te_ = 0.073, MAPE_te_ = 0.16), this means that the screening of tea germplasm resources with drought tolerance can be completed before there is no obvious phenotypic change in the aboveground part of tea germplasm resources. Therefore, using the hyperspectral camera to screen tea germplasm resources with drought tolerance is an efficient method. The model not only achieves the expected effect but also has high prediction accuracy. Through the research and application of this model, the identification and evaluation of tea germplasm resources with long seedling stage and small phenotypic change can be realized, so as to accelerate the artificial breeding process of drought resistant tea germplasm resources.

## Data availability statement

The raw data supporting the conclusions of this article will be made available by the authors, without undue reservation.

## Author contributions

SC, carried out the experiment, collected and organized data, processed the hyperspectral image of tea leaves and wrote the manuscript. JS, KF and WQ participated in designing the experiment and reviewed the manuscript. ZD and YW, raised the hypothesis underlying this work, designed the experiment, and helped organize the manuscript structure and directed the study. HG, YL, JZ and XH participated in designing the experiment and directed the study. All authors contributed to the article and approved the submitted version.

## Funding

This research was funded by the Innovation project of Shandong Academy of Agricultural Sciences (CXGC2022E18, CXGC2022B03); the Technology System of Modern Agricultural Industry in Shandong Province (SDAIT-19-01); The Special Foundation for Distinguished Taishan Scholar of Shandong Province (No. ts201712057); the Rizhao science and technology innovation project (2020cxzx1104); Qingdao people's livelihood plan (21-1-4-ny-2-nsh).

## Conflict of interest

The reviewer JZ declared a shared affiliation with the author JS to the handling editor at the time of review.

The authors declare that the research was conducted in the absence of any commercial or financial relationships that could be construed as a potential conflict of interest.

## Publisher’s note

All claims expressed in this article are solely those of the authors and do not necessarily represent those of their affiliated organizations, or those of the publisher, the editors and the reviewers. Any product that may be evaluated in this article, or claim that may be made by its manufacturer, is not guaranteed or endorsed by the publisher.
