# Supplementary material for: Hyperspectral machine-learning model for screening tea germplasm resources with drought tolerance
Source: Front Plant Sci. 2022 Dec 1;13:1048442. doi: 10.3389/fpls.2022.1048442 (PMC9751484; doi:10.3389/fpls.2022.1048442)
Supplement: Supplementary file 1 [file Table_1.docx]

Supplement Table 1 Variety name and full name

| Variety name | Full name of variety |
| --- | --- |
| *‘SCZ'* | Su-chazao |
| *‘ZC 108'* | Zhongcha 108 |
| *‘MS 9'* | Mengshan 9 |
| *‘QN 1'* | Qingnong 1 |
| *‘QN 21'* | Qingnong 21 |
| *‘QN 36'* | Qingnong 36 |
| *‘QN 38'* | Qingnong 38 |
| *‘JGY'* | Jin-guanyin |
| *‘JX'* | Jinxuan |
| *‘XY 10'* | Xinyang 10 |

| Index | Varieties | MAX | MIN | STDEV | AVERAGE | CV |
| --- | --- | --- | --- | --- | --- | --- |
| MDA | *‘SCZ'* | 22.2366 | 7.3128 | 4.052711 | 12.83838 | 0.315672 |
|  | *‘ZC 108'* | 18.1512 | 7.6306 | 3.181556 | 12.1746 | 0.261327 |
|  | *‘MS 9'* | 18.2288 | 8.398 | 2.492457 | 11.9581 | 0.208433 |
|  | *‘QN 1'* | 18.482 | 6.367 | 3.194541 | 11.57142 | 0.276072 |
|  | *‘QN 21'* | 17.898 | 8.0466 | 2.996394 | 12.23764 | 0.244851 |
|  | *‘QN 36'* | 20.4122 | 7.8166 | 3.02092 | 11.47821 | 0.263187 |
|  | *‘QN 38'* | 20.0272 | 7.2352 | 3.718834 | 12.03764 | 0.308934 |
|  | *‘JGY'* | 17.4562 | 7.0892 | 2.736524 | 12.86785 | 0.212664 |
|  | *‘JX'* | 22.818 | 7.2998 | 3.782319 | 13.13631 | 0.287928 |
|  | *‘XY 10'* | 17.4122 | 7.6874 | 2.609819 | 12.04797 | 0.216619 |
| SS | *‘SCZ'* | 45.02034 | 9.33994 | 8.579028 | 19.44512 | 0.441192 |
|  | *‘ZC 108'* | 36.28054 | 10.86334 | 6.60305 | 22.64535 | 0.291585 |
|  | *‘MS 9'* | 38.86534 | 15.81754 | 6.413752 | 25.93002 | 0.247348 |
|  | *‘QN 1'* | 34.34194 | 15.17134 | 5.125214 | 25.10835 | 0.204124 |
|  | *‘QN 21'* | 40.15774 | 13.87894 | 6.761851 | 24.13568 | 0.28016 |
|  | *‘QN 36'* | 36.28054 | 13.47934 | 6.816582 | 24.72695 | 0.275674 |
|  | *‘QN 38'* | 37.14214 | 9.35554 | 7.726541 | 23.861 | 0.323815 |
|  | *‘JGY'* | 42.09634 | 8.49394 | 9.382182 | 21.25621 | 0.441385 |
|  | *‘JX'* | 42.74254 | 8.26294 | 9.258193 | 21.92988 | 0.422173 |
|  | *‘XY 10'* | 43.81954 | 6.55534 | 10.21572 | 24.11688 | 0.423592 |
| TP | *‘SCZ'* | 7.11684 | 3.25144 | 1.029939 | 4.817526 | 0.21379 |
|  | *‘ZC 108'* | 8.31104 | 1.77444 | 2.014583 | 5.492011 | 0.366821 |
|  | *‘MS 9'* | 8.35704 | 1.58364 | 2.006079 | 4.442011 | 0.451615 |
|  | *‘QN 1'* | 7.88004 | 1.69284 | 1.573334 | 4.56099 | 0.344955 |
|  | *‘QN 21'* | 6.63984 | 1.01124 | 1.369275 | 3.80969 | 0.359419 |
|  | *‘QN 36'* | 8.73864 | 3.82384 | 1.22498 | 5.308561 | 0.230755 |
|  | *‘QN 38'* | 7.73864 | 1.86984 | 1.484416 | 3.99109 | 0.371932 |
|  | *‘JGY'* | 9.20884 | 2.29744 | 2.126084 | 5.561419 | 0.382292 |
|  | *‘JX'* | 6.83064 | 1.67904 | 1.336818 | 4.261897 | 0.313667 |
|  | *‘XY 10'* | 7.59384 | 3.63304 | 1.101426 | 5.087583 | 0.216493 |

Supplement Table 2 Physiological and biochemical data of different tea germplasm resources measured with the kit
